# Connected diagnostics to improve accurate diagnosis, treatment, and conditional payment of malaria services in Kenya

**DOI:** 10.1186/s12911-021-01600-z

**Published:** 2021-08-04

**Authors:** Shannen M. C. van Duijn, Angela K. Siteyi, Sherzel Smith, Emmanuel Milimo, Leon Stijvers, Monica Oguttu, Michael O. Amollo, Edward O. Okeyo, Lilyana Dayo, Titus Kwambai, Dickens Onyango, Tobias F. Rinke de Wit

**Affiliations:** 1 PharmAccess Foundation, Amsterdam, The Netherlands; 2 PharmAccess Foundation, Nairobi, Kenya; 3 Kisumu Medical and Education Trust (KMET), Kisumu, Kenya; 4 Nightingale Hospital, Kisumu, Kenya; 5 Malaria Control Program Coordinator-Kisumu County – Ministry of Health, Kisumu, Kenya; 6 Kenyan Medical Research Institute (KEMRI), Kisumu, Kenya; 7 County Department of Health, Kisumu, Kisumu County Kenya; 8 Joep Lange Institute, Amsterdam, The Netherlands

**Keywords:** Diagnosis, Treatment, Conditional payments, Malaria, Kenya, Connected diagnostics

## Abstract

**Background:**

In sub-Saharan Africa, the material and human capacity to diagnose patients reporting with fever to healthcare providers is largely insufficient. Febrile patients are typically treated presumptively with antimalarials and/or antibiotics. Such over-prescription can lead to drug resistance and involves unnecessary costs to the health system. International funding for malaria is currently not sufficient to control malaria. Transition to domestic funding is challenged by UHC efforts and recent COVID-19 outbreak. Herewith we present a digital approach to improve efficiencies in diagnosis and treatment of malaria in endemic Kisumu, Kenya: Connected Diagnostics. The objective of this study is to evaluate the feasibility, user experience and clinical performance of this approach in Kisumu.

**Methods:**

Our intervention was performed Oct 2017–Dec 2018 across five private providers in Kisumu. Patients were enrolled on M-TIBA platform, diagnostic test results digitized, and only positive patients were digitally entitled to malaria treatment. Data on socio-demographics, healthcare transactions and medical outcomes were analysed using standard descriptive quantitative statistics. Provider perspectives were gathered by 19 semi-structured interviews.

**Results:**

In total 11,689 febrile patients were digitally tested through five private providers. Malaria positivity ranged from 7.4 to 30.2% between providers, significantly more amongst the poor (*p* < 0.05). Prescription of antimalarials was substantially aberrant from National Guidelines, with 28% over-prescription (4.6–63.3% per provider) and prescription of branded versus generic antimalarials differing amongst facilities and correlating with the socioeconomic status of clients. Challenges were encountered transitioning from microscopy to RDT.

**Conclusion:**

We provide full proof-of-concept of innovative Connected Diagnostics to use digitized malaria diagnostics to earmark digital entitlements for correct malaria treatment of patients. This approach has large cost-saving and quality improvement potential.

**Supplementary Information:**

The online version contains supplementary material available at 10.1186/s12911-021-01600-z.

## Background

Sub-Saharan Africa (SSA) continues to face challenges attaining accurate diagnosis of both infectious and non-communicable illnesses. Laboratory diagnosis remains suboptimal with much-needed lab equipment often lacking, financial resources being scarce and skilled medical staff underrepresented [1]. Recently, across 49 SSA countries, only 380 laboratories were accredited against international standards, with many countries not even hosting one single accredited provider [2]. Also, in urban and semi-urban centres where lab facilities and staff are usually better available, inaccurate diagnoses with limited sensitivity and specificity are common practice [1, 3–5]. Fever is one of the most common reasons people in Africa visit health providers. However, due to the aforementioned challenges with lab providers across the continent, febrile patients are often not diagnosed using laboratory tests, but only presumptively, based on clinical grounds [1]. The most common presumptive diagnosis in febrile patients is malaria, followed by bacterial infection(s), resulting in over-prescription of antimalarials and antibiotics. A recent study indicated that more than 70% of fever cases in Tanzanian children was caused by viral infections, against which antimalarials and antibiotics do not work [6].

SSA countries hold a disproportionately high share of the global malaria burden. In 2018, the region hosted 93% of malaria cases and 94% of malaria deaths [7]. Over 99% of malaria cases in malaria-endemic areas of Africa were caused by *P. falciparum* in 2018 [7]. Substantial investments continue to be needed to reduce malaria morbidity and mortality in Africa. Estimates show that by 2025, the global annual malaria investments should increase to $7.7 billion [8]. However, global funding for malaria control and elimination in Africa is flat-lining and the recent call for universal health coverage (UHC) puts pressure on traditional vertical funds for malaria, such as the Global Fund to Fight Aids Tuberculosis and Malaria (GFATM) and President’s Malaria Initiative (PMI) [9, 10]. Additional challenges to malaria service delivery are emerging with the recent outbreak of COVID-19 [11]. Important drawbacks in combatting malaria were encountered in West Africa during the recent Ebola outbreak, setting examples for responding to the imminent COVID-19 pandemic [12], indicating the importance of more efficient, targeted digital malaria service delivery.

Presumptive diagnosis and treatment for malaria in febrile patients often lead to over-prescription of malaria drugs in malaria-endemic African countries [13, 14]. This can have key health implications, including the development of drug resistance, higher risk of treatment failure, and increased morbidity. Besides, it leads to economic implications such as unnecessary drug costs, recurring visits, and economic productivity losses due to longer sick days. Consequently, the costs for both households and healthcare providers increase [15, 16]. Moreover, the prescription of ineffective drugs can lead to reduced patient trust in healthcare provision with subsequent decreased willingness to participate in financial (insurance) schemes for pre-payment and risk-sharing.

Recent developments in digital technology provide new opportunities to address the above situation in a radically different way. Firstly, the emergence of the Internet of Things comprising a rapidly growing arsenal of ‘digital diagnostics’: tools and devices that digitalize and link human physical parameters to the internet, complete with sensing and measuring capabilities [17]. Secondly, the mobile revolution: 75% of the African population has access to a mobile phone. In Kenya, mobile penetration is at 86%, and still growing rapidly [18, 19]. Thirdly, Africa is leapfrogging with the advent of ‘bankless banking’: digital payment systems through mobile phones transferring entitlements between individuals. An example of this is M-PESA, launched in 2007 in Kenya: an electronic mobile money service to store, send and receive money on any mobile phone (smartphone or non-feature) with an M-PESA account [20].

Since 2014, the non-profit foundation PharmAccess has leveraged the above developments to create a ‘mobile health wallet’ linked to the M-PESA payment platform, known as M-TIBA (‘mobile therapy’ in Swahili). Today, M-TIBA is hosted by CarePay International and represents the first African digital platform exchanging data and funds/entitlements that are exclusively earmarked for health and healthcare [21]. M-TIBA works on simple, non-feature mobile phones as well as smartphones. It connects patients, healthcare providers and healthcare payers (such as insurers and donors) and exchanges data and entitlements between them. Users can save into their own (family) wallets, they can receive money from relatives elsewhere in the country, from donors, and even from individuals in other countries willing to donate directly for health [22]. This is a first step in creating new digital solidarity mechanisms where people can financially contribute to each other’s health: the rich for the poor, the healthy for the sick, the young for the old, and communities for individuals. As of April 2020, 4.2 million people in Kenya, Nigeria, Tanzania and 1500 health providers on the continent were connected to the platform [21].

Given the above developments, we saw an opportunity within the Kenyan context to combine digital diagnostics and M-TIBA technology to support marked improvements in the efficiency of diagnosis and treatment of febrile diseases. This approach is referred to as ‘Connected Diagnostics (ConnDx)’. ConnDx introduces a new system-wide digital delivery approach for malaria care, linking phones to diagnostics and payment systems. Providing ConnDx services yields simultaneous real-time insight into data and funds exchanged between patients, providers, and healthcare payers.

ConnDx digitalizes rapid diagnostic tests (RDTs) through photography and interprets and stores results in cloud-based databases through dedicated RDT readers. Such readers are available, like the Fio Deki Reader™ [23], i-CalQ lateral flow-test imaging [24] and the Mobile Assay ‘lab-on-mobile device’ platform [25]. Moreover, some Apps can directly digitalize RDT results [26]. The digital diagnostic results subsequently inform a mobile phone-based payment system (M-TIBA) to channel dedicated funds for diagnosis and treatment of the pertinent disease to the provider and/or patient. A recent proof-of-principle of ConnDx was provided in Samburu, Kenya, in which RDTs interpreted by the Fio Deki Reader™ were used to diagnose brucellosis in remote populations [27]. Brucellosis, a rare bacterial zoonotic illness, is often underdiagnosed due to having similar febrile symptoms as malaria [28]. Patients testing positive were directly linked to funds through their respective mobile health wallets to allow for payment of drugs for the pertinent diseases, conditional to a positive diagnosis of these diseases. Feasibility was demonstrated, patients were correctly treated, their user experience was positive, and hotspots of brucellosis were identified [27].

Having proven that ConnDx could be effective in identifying a rare disease in rural, remote populations, this paper reports on the next step: scaling ConnDx to a more densely populated area for a more prevalent disease (malaria) that causes high morbidity, mortality and serious economic impact. The objective of this study is to evaluate the feasibility, user experience, and clinical performance of a ConnDx intervention in Kisumu, Kenya. Additionally, the aim is to assess the over-prescription of antimalarials in this population. The location of choice, Kisumu County in Kenya, has a population of 1.2 million [29], and a reported high malaria prevalence of approximately 28–35% in the general population [30, 31]. Our intervention took place from October 2017–December 2018 amongst five private health providers and a total of ~ 12,000 beneficiaries. The private sector market share for malaria is large in Kisumu and the whole of Kenya. The majority (three-fourths) of antimalarials are distributed through private sector providers [32].

## Methods

The ConnDx process starts upon patient presentation at participating providers or healthcare outreach events. The project was announced to the public through posters in the waiting room referring to a free ‘Malaria Test & Treat Campaign’. Consenting patients presenting with fever and/or malaria symptoms were referred by informed clinicians for malaria testing by qualified lab technicians using malaria RDTs, digitalized using a Fio Deki Reader™. The Reader contained a drop-down menu to collect demographic data (gender, age, pregnancy status), basic economic data (socioeconomic status) and geographic location data (community of residence). For socioeconomic status assessment, three questions were added to the menu pertinent to access to electricity, toilet type and education level of the household head. These questions had been previously identified most informative by principal component analysis using a Multiple Indicator Cluster Survey in Nyanza for reference [33]. This led to a poverty index, in which level 1 corresponds to the poorest category; level 3 to the least poor. With geographical location data, malaria hotspots can be identified. In this context, we define a hotspot as a geographical area in which the malaria transmission intensity is significantly higher than the surrounding area.

**Fig. 1:**
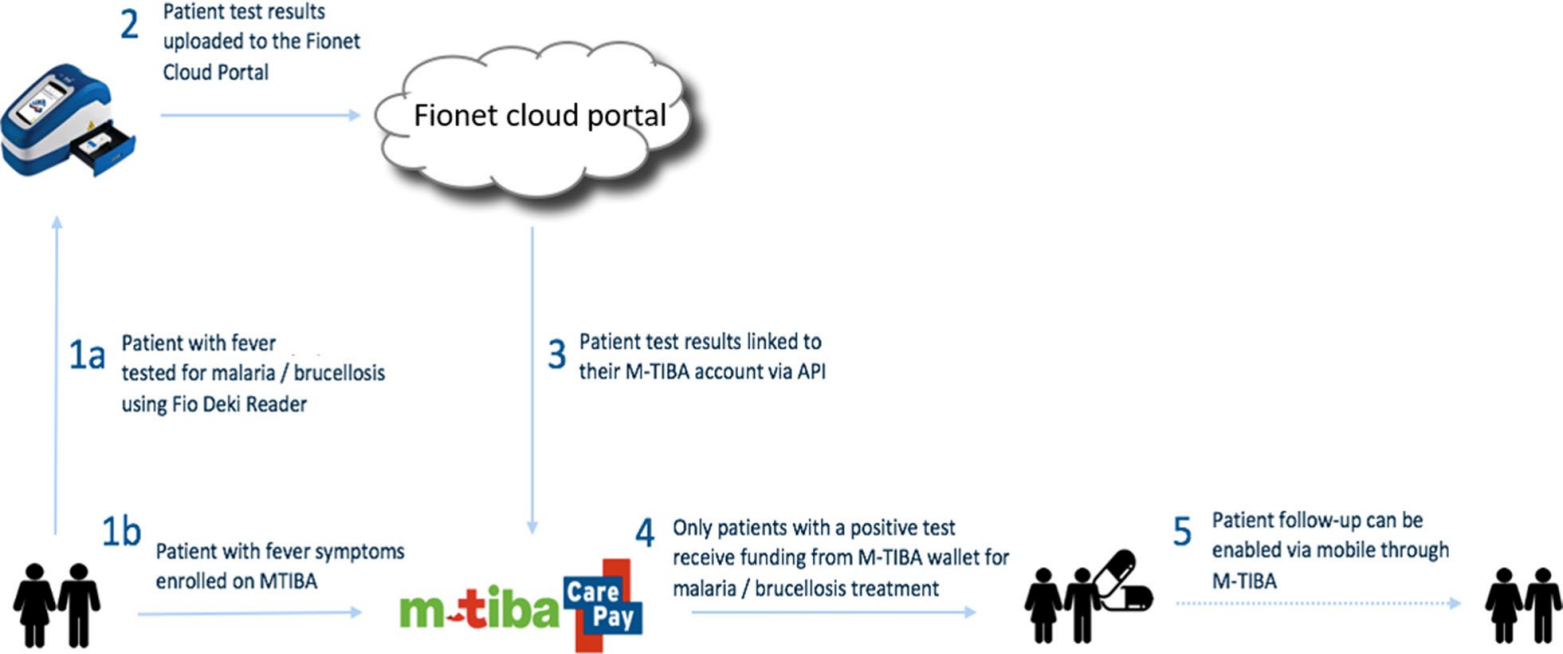
Schematic illustration ConnDx process. Step (1) Patient with fever symptoms is tested for malaria and enrolled on M-TIBA. (2) Test results of patient are uploaded to the cloud portal of Fionet. (3) The test results are also linked to the M-TIBA account of the patient. (4) Patients with a positive test receive funding for treatment. (5) Patient follow-up can be enabled via mobile through M-TIBA

Simultaneously, the participating patients were enrolled on the M-TIBA platform and individual health wallets were created. The collected Fio Deki Reader™ demographic data, along with patient test results, was uploaded to the Fionet cloud database and linked to the patient’s mobile health wallet account using a unique M-TIBA transaction code. Only patients with a positive diagnosis were entitled to receive funding for antimalarials, which (in an interim functionality) was paid to their respective healthcare providers, thus effectuating fully paid treatment for the patients. Patients received antimalarials at the same provider as where they received a positive diagnosis. Figure 1 provides a schematic illustration of the ConnDx process.

ConnDx was implemented from October 2017–December 2018 across five private care providers (anonymized A-E) within sub-counties of Kisumu County. Providers were selected based on DHIS-2 registered (high) patient throughput, geographic location close to water, the willingness of management to participate and ranged from smaller providers with set opening hours to large 24/7 hospital facilities.

The provider perspectives on the use of ConnDx were gathered through 19 semi-structured interviews in the participating health providers. Interviewees were selected in agreement with the facility manager or based availability of staff members at the time of the interviews. Interviewed staff were managers, lab technicians, receptionists, and doctors or nurses. Informed consents were obtained from each interviewee before the interview. The interview guide, specifically developed for this study, covered topics related to acceptability of the intervention and outstanding outcomes of the quantitative data analysis that needed further clarification (Additional file 1). In total, four types of interview guides were constructed, based on the professional background of the respondents. The first interview held served as a pilot interview to test the interview guide. The interviews took place at the work location of the interviewees. One PharmAccess researcher held, transcribed, coded, and analyzed the interviews. The first five interviews were coded openly. Thereafter, all codes were compared to each other and one list of codes was created that was used to code the remaining 14 interviews. The interviews were analyzed by applying a thematic analysis approach using the software Atlas.ti [34]to identify patterns and themes within the data. Two other researchers checked the coding and theme patterns to ensure consistency in the analysis of the interviews.

Quantitative data from both cloud-based databases (FIO and M-TIBA) were analyzed through descriptive statistics in Stata 12 and Microsoft Excel 2016. Descriptive analyses were done for each provider separate and over time. Relationships between categorical variables were tested by using the chi-square test to calculate *p* values and compare proportions between groups. A *p* value of < 0.05 was considered statistically significant. For geographic location analyses, the subdivision of Kisumu County was divided into the 163 administrative community units, each with roughly 1000–1200 households. Participants were asked at the point of care which community unit their home was based. Geographic analyses were performed in QGIS (Version 3.0) [35] by using the geographic centre of each community unit as a proxy for the location of the participants’ domicile. The project was positioned as a quality improvement project of existing malaria services according to Kenyan Guidelines and run in parallel to existing systems and services. All study participants provided consent for participation before the enrolment process into M-TIBA.

## Results

**Fig. 2:**
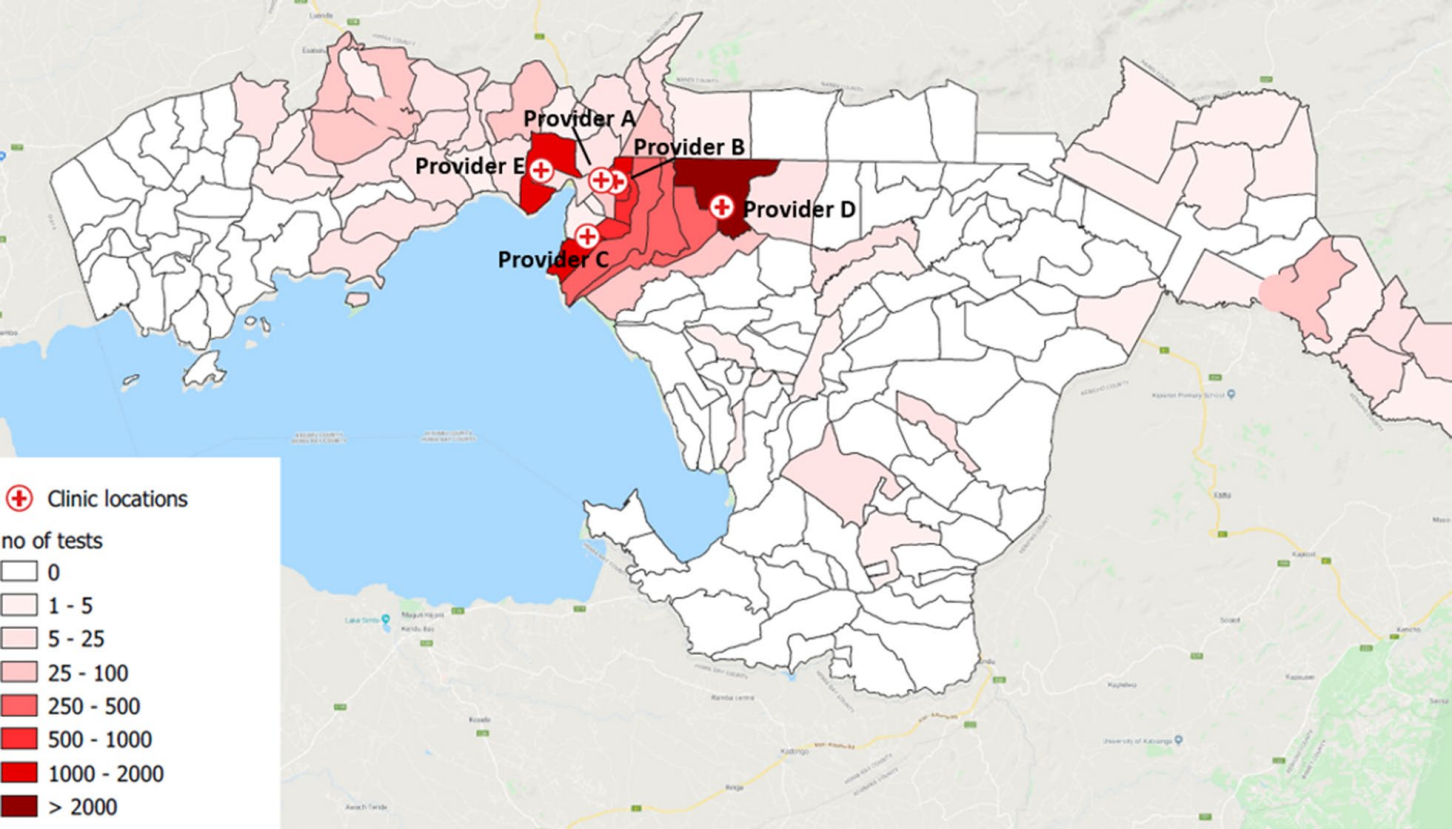
Location of the participating providers A–E in Kisumu County and hotspots of malaria tests. Data used to construct this map is gathered during this intervention. This map and the layers presenting the data on the map were created using location data from our dataset using QGIS software

Between October 2017 and December 2018, 11,689 people with fever were tested in five private providers (A-E). Figure 2 depicts the numbers of malaria RDTs uploaded by location of these providers and participants’ domiciles according to the community unit they reported to live in.

**Fig. 3:**
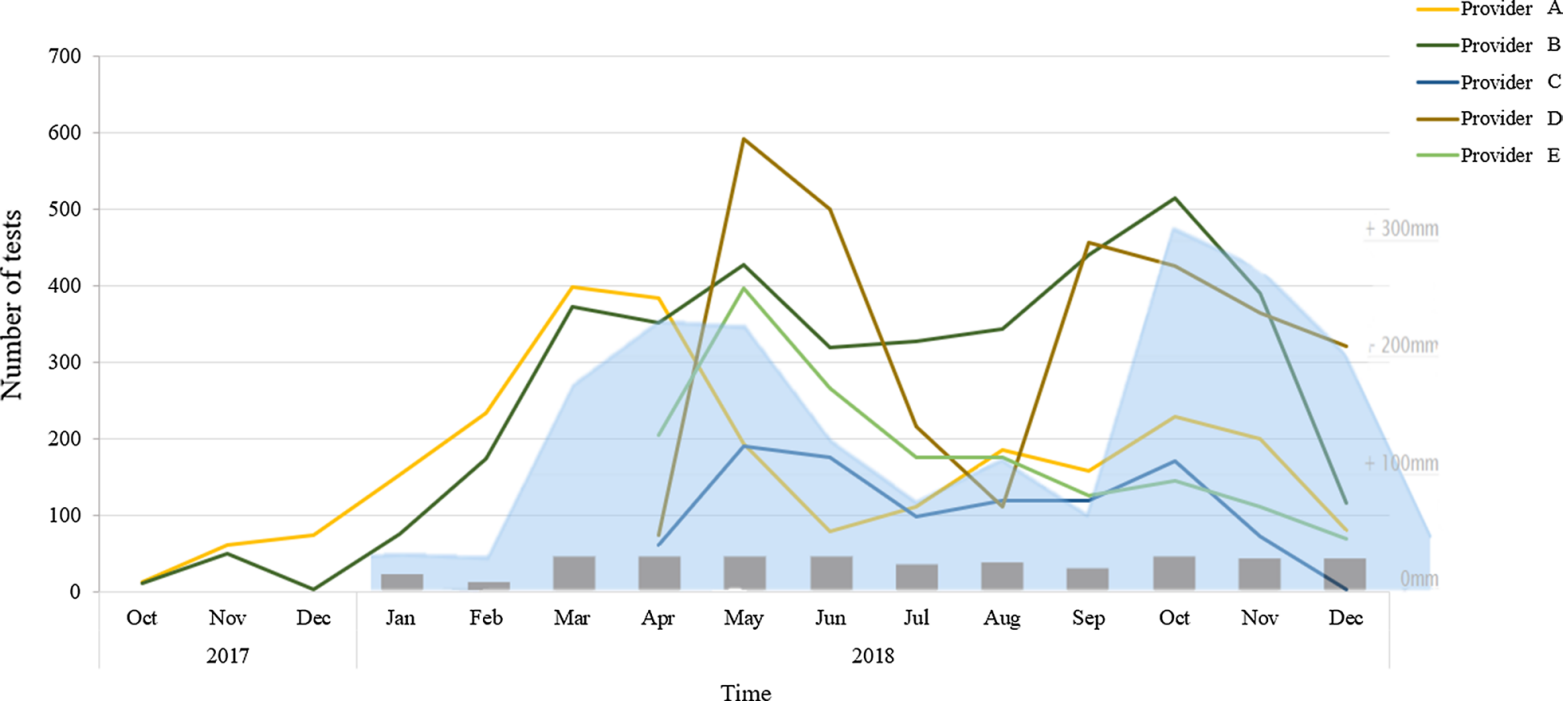
Participation dynamics providers A–E during the pilot of ConnDx in Kisumu. Number of transactions (tests) per provider from October 2017–December 2018 derived from FIO database. Monthly rainfall in 2018 is indicated grey bars per months and in the light blue graph. Derived from worldweatheronline.com

Figure 3 shows the participation dynamics per provider during the intervention. Providers A and B started with ConnDx in October 2017, providers C, D and E enrolled in April 2018. Malaria testing rates coincided with the rainy seasons in Kisumu for 2017 and 2018 (“long rains” from March to June, and “short rains” from October to December), known to increase malaria transmission. During the study period (2018), rainfall in Kisumu was particularly high and prolonged compared to the average [36].

We found a variation between the providers regarding positivity rates of malaria tests. Provider B reported relatively the least positive malaria tests (7.4%; 290/3908). This is followed by provider C (12.6%; 127/1006), provider E (16.7%; 279/1666) and provider A (18.1%; 460/2548). Provider D identified the highest malaria positivity rates (30.2%; 922/3057).

From all 11,689 consenting patients who were tested for malaria, there was an 18.3% overall test positivity rate. The descriptive analyses showed that slightly more women were tested (58.2%), but more positive cases were found amongst men (19.0% versus 15.4%). The mean age of patients was 23.6 years and 16.5% of patients were aged under 5 years old. For children under 5 years of age, the percentage of positive malaria cases was comparable to adults 17.7% (n = 340/1926). From the participants, 63.1% were a member of NHIF, the national health insurance in Kenya. A chi-square test showed patients in poverty level 1 were significantly more often tested positive for malaria than patients with a higher poverty score (*p *value < 0.05).

**Fig. 4:**
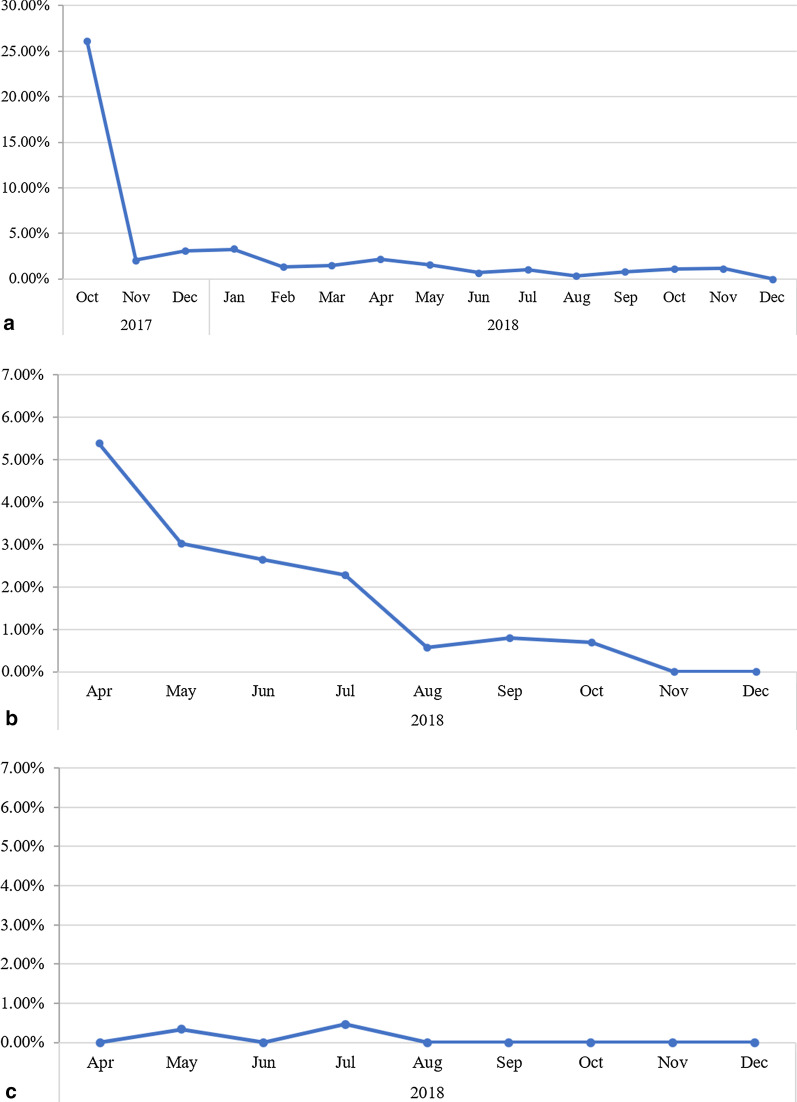
**a** Overall percentage of RDTs with errors, from October 2017–December 2018. **b** Provider E percentage of RDTs with errors, from April 2018–December 2018 **c** Provider D percentage of RDTs with errors, from April 2018–December 2018

The RDT result uploads of ConnDx allowed for quality assessment since the Deki Reader™ indicates ‘error’ at point of care when the RDT is not performed correctly. Figure 4a–c illustrate the quality improvement of lab technicians’ RDT performance represented by reductions of error rates over time. Figure 4a demonstrates considerable overall improvement over the first month of RDT usage (from 25% range down to below 5%). Figure 4b, c provide individual data for later starters E (improvement) and D (continued good performance).

**Fig. 5:**
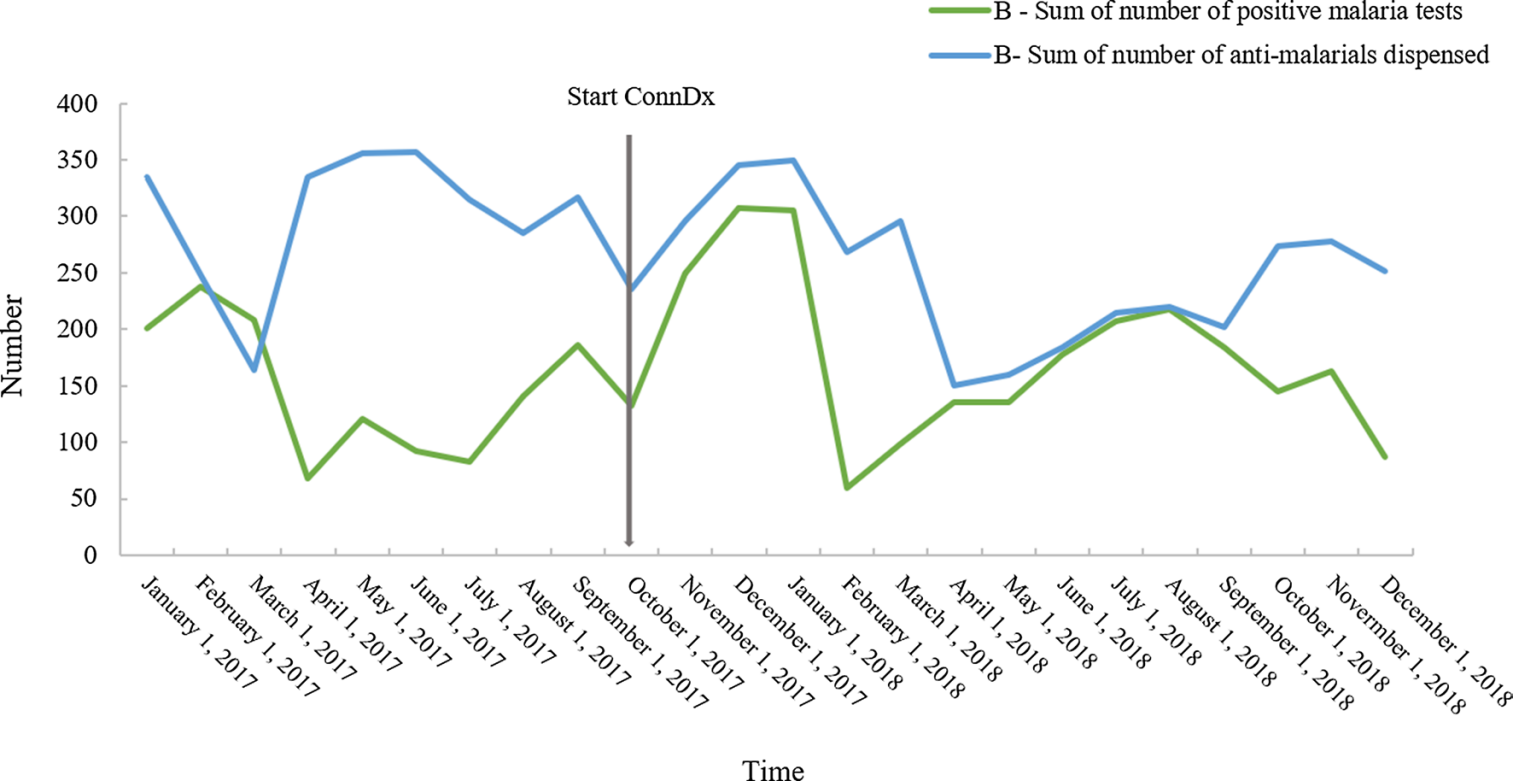
Number of positive malaria tests compared with number of antimalarials prescriptions dispensed within provider B throughout the project and the 9 months prior to implementation

For all participating providers, general over-prescription of antimalarials was observed. Over-prescription is defined by the proportion of antimalarials dispensed to the actual number of positive cases as identified by a positive malaria test. The overall over-prescription was 28.0%, fluctuating between 4.6% (provider D) and 63.3% (provider B). There were fluctuations in over-prescription over time, as illustrated in Fig. 5. High over-prescription rates were recorded before the introduction of ConnDx at provider B. During the ConnDx intervention, over-prescription declined significantly. Only towards the end of the intervention, an increase was observed.

**Fig. 6:**
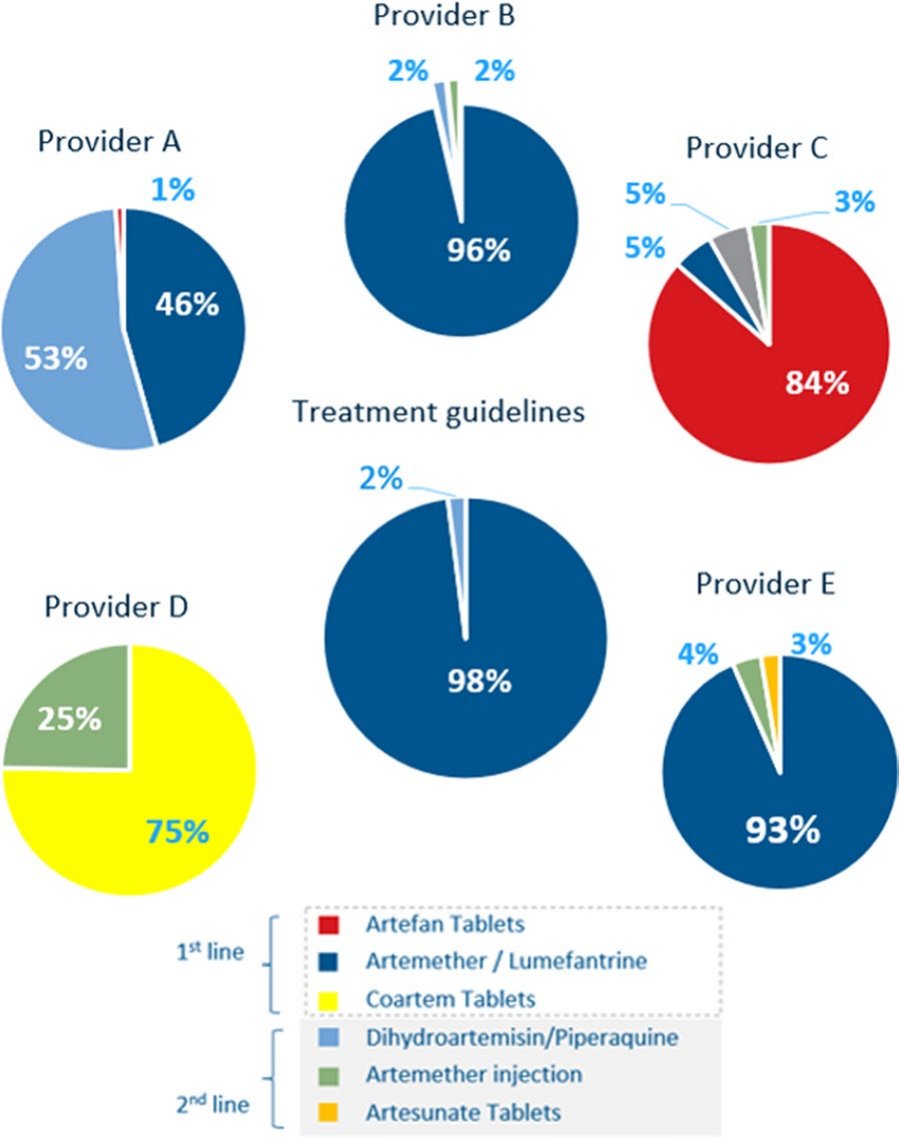
Type of antimalarials dispensed during ConnDx pilot per provider compared to the treatment guidelines (pie diagram in the middle), divided in 1st versus 2nd line

Figure 6 demonstrates M-TIBA derived information on individual provider-specific prescribing behaviour in choices of drugs (1st line and 2nd line) for antimalarials. Descriptive analyses showed the most common prescriptions were: artemether/lumefantrine (75.4%, either branded (Coartem, 43.7%) or generic (28.5%), followed by artemether injection, a 2nd line drug (15%). Overall, 75.6% of all antimalarials dispensed by the providers were 1st line and 24.4% were 2nd line. It was found that 2nd line antimalarials were significantly more frequently prescribed to the more affluent population and to participants who were not insured with NHIF (*p* value < 0.05; chi-square test). Provider A dispensed most 2nd line antimalarials (average 53.7, ranging from 26.5%—100.0%). This is followed by provider D (24.7%), provider B (9.2%), provider E (7.1%) and provider C (3.9%). Figure 6 illustrates that three (A, C, D) out of five private providers substantially diverted from the Kenyan guidelines for the prescription. The choice of branded versus generic antimalarials appeared dichotomous: providers A, B and E prescribed predominantly generics and providers C and D branded drugs.

Qualitative interviews with the healthcare providers identified various perceived challenges and opportunities. Concerning opportunities, ConnDx malaria diagnosis through RDT was experienced as easier, more efficient, and faster. Providers mentioned patients were also positive towards this way of testing for malaria which qualified them to receive payments for treatment. Moreover, healthcare providers mentioned ConnDx providing interesting additional (management) information on patients, prescription practices and drug procurement. The interviewees appreciated ConnDx to expand access to malaria treatment, particularly for the poor patients and for children. Providers experienced improvement on their awareness that diagnostics should determine treatment decisions, reducing the prescriptions of unnecessary drugs.

One of the challenges mentioned by healthcare providers was a lack of trust in the performance of RDTs. Microscopy is still often seen as the gold standard for malaria testing in Kenya, despite National Guidelines indicating equality of RDT and microscopy as a diagnostic procedure. Some healthcare providers mentioned they verified RDT results by microscope. Other challenges were more technical, related to lack of electrical power and internet connection. Despite these challenges, most respondents stated they would continue with ConnDx, as it did help many patients.

*“It was positive. Because for us being in the community and a malaria-prone area, it was part of the solution that we have been wanting. So, we really embraced it, it was positive.”*—Respondent 18 (admin, female).

## Discussion

This study describes feasibility and user experience through private healthcare providers in Kisumu of a novel digital approach to malaria diagnosis that directs conditional payments for malaria treatment: ConnDx. We demonstrate significant potential for increasing efficiencies of malaria service delivery in the Kenyan private healthcare sector concerning better diagnosis, reducing over-prescription, selecting correct 1st and 2nd line drug combinations and reducing malaria transaction costs, while at the same time generating valuable real-time data on malaria prevalence and incidence that can be fed into the DHIS-2, that captures routine health service data.

ConnDx proved through this pilot its potency to monitoring malaria epidemiology in semi-real time and generate important data for malaria management. Considerable variation was revealed between providers, with malaria positivity rates ranging from 7.4% (provider B) to 30.2% (provider D). This led to verifiable assumptions such as Provider B being a referral hospital and therefore less likely to serve primary malaria cases, while provider D, being located near wet rice fields, serving known hotspots for malaria. During months with more rainfall, there were significantly more malaria tests done at the providers than months with less rainfall (*p* value < 0.05, chi-square test). However, no relation was found between months with more rainfall and positive malaria test results. Providers located in or near low-income settlements (A and E) appeared to have higher malaria positivity rates. A chi-square test showed a relation between poorer patients and positive malaria tests (*p* value < 0.05, chi-square test). The quantitative analyses showed a relatively low participation rate of children: only 16.5% of reported patients were aged < 5 years, with a positivity rate of 17.7%. Through our qualitative interviews, it was learnt that more children were tested for malaria, but clients experienced challenges subscribing their children to M-TIBA as dependents and sometimes reported them incorrectly as adult primary members. This was noticed later during the campaign and corrected but could have contributed to general underreporting of pediatric malaria cases. All in all, it was demonstrated that ConnDx can facilitate in semi-real time important healthcare provider-differences in malaria case management. Such information, when collected at a larger-scale level could help policymakers and health system managers to target their efforts for (human) malaria capacity building.

Secondly, this study demonstrated the overall potency of ConnDx to monitor provider prescription behaviours and identify practices that are significantly aberrant from the Kenyan National Guidelines. Important overall over-prescription was recorded of antimalarials (28.0%), varying between providers from 4.6% (provider D) to 63.3% (provider B). There are multiple reasons for over-prescription, ranging from monetary considerations of private providers to patient expectation and pressure to receive drugs, avoidance of clinicians to take the risk of a false negative diagnosis and subsequent fatality, etc. [14]. Our qualitative interviews showed patient pressure was mentioned as a reason by most of the interviewees when addressing this issue. Furthermore, ConnDx revealed an unexpected and erroneously high level of prescription of 2nd line antimalarials (overall 28.0%). Provider A revealed 2nd line prescription levels of overall 53.7%, at times going up to even 100%. This is remarkable, as 2nd line antimalarials are generally used for severe cases of malaria, which represent on average < 2% [37], or in (rare) cases of suboptimal parasitological response with 1st line antimalarials (resistance). When probed with this observation, provider A reported a prolonged stock-out of 1st line antimalarials and therefore switching to 2nd line. Over-prescription of 2nd line antimalarials was more often found with more affluent and uninsured participants. This could indicate providers are aware of the socioeconomic status of their clients and they incorporate this into their prescriptions. Moreover, it appeared there was a very dichotomous, almost exclusive usage of either branded (provider C and D) or generic (provider A, B, E) antimalarials. One possibility could be that providers serving more affluent customers prefer procurement of branded versus generic antimalarials. Conversely, more affluent customers might request for branded instead of generic antimalarials. Often, generic medicines are considered to be of poor quality and treated with more suspicion than branded medicines [38, 39].

Third, this study indicates that ConnDx can increase efficiency in malaria service delivery by decreasing costs in several ways. Over-prescription of antimalarials can be monitored, aberrations identified, and actions can be undertaken to address those. A 2013 study conducted in four providers in western Kenya, noted that presumptive malaria treatment can lead to misdiagnosis rates as high as 53% in public facilities. [13]. ConnDx can play an important role to reduce such a figure in facilities. Further cost reductions can potentially be realized by ConnDx, such as decreasing paperwork in health providers; such automated systems saving time, manpower and being more accurate in reporting cases [40]. In addition, ConnDx implies less dependency on expensive and maintenance-dependent microscopy, and less electricity will be required to perform diagnostics tests. Moreover, due to its user-friendliness ConnDx, will provide more opportunities for lower trained lab-staff to perform such tests, saving personnel costs.

Finally, and most importantly, ConnDx can facilitate a much more targeted bottom-up payment for malaria services to providers and clients, creating unprecedented transparency as compared to current top-down payment systems [manuscript in preparation]. During this pilot, patients also benefitted from the reduction in costs as they received a free RDT test and treatment. However, when these would be obtained in a private facility outside this pilot, patients would have to pay on average 150 KES ($1.38 at the time) for an RDT and 150–500 KES ($1.38–$4.59 at the time) for malaria treatment when tested positive, depending on whether they require first line or 2nd treatment. In public facilities, the average costs to patients per malaria visit were found to be 112 KES ($1.03) in 2016, including registration fees, diagnosis, and treatment [41]. However, in public facilities, there is a gap in the availability of both testing and treatment of malaria [32].

Overall user experiences of ConnDx from the perspectives of providers were positive. For all providers, the key challenge was reservations of their staff to adopt the use of RDTs instead of microscopy. In general, microscopy was still seen as the golden standard for malaria testing in Kenya, despite National Guidelines indicating equality of RDT and microscopy as diagnostic procedure [42]. The fact that microscopy can identify different malaria species and quantify the severity of infection is counterargued by the fact that 1st line treatment for uncomplicated malaria is identical for Eastern African malaria species (see below), independent of their load.

Indeed, ConnDx is dependent on the use of RDTs instead of microscopy for malaria diagnostic testing. Apart from National Guidelines, also the international literature reports sensitivity and specificity of malaria RDTs equal to microscopy [43, 44]. RDTs are also recommended by WHO [3]. Several studies demonstrated impaired sensitivity of microscopy in actual field situations in Africa as compared to perfectly controlled laboratory circumstances, with regular refresher training being required [33, 34]. RDTs have the added advantage that, in contrast to microscopy, these can easily be externally quality controlled by visual inspection of independent third parties. This opportunity is further enhanced through the ConnDx feature of making digital photographs of every test result, stored in secure cloud-based databases that can be accessed anywhere in the world. An additional advantage of RDT is that results can be digitalized, which accelerates data collection to (semi)real-timeliness, allows for telemedicine-based quality control and improves quality and completeness of data collection (versus paper-based malaria files being entered into national DHIS-2 systems on a several-time-per-year basis). These options are all much more problematic when performing microscopy. Moreover, in the sub-Saharan African reality, RDTs can readily detect *Plasmodium falciparum*, which causes the highest malaria morbidity and mortality and represents 99.7% of cases [35]. RDTs indeed are less available that specifically detect *P. vivax*, but this species is virtually absent in the region. Finally, indeed RDTs can provide a false-positive result with patients who had recent malaria episodes. This can be addressed by building a feature into the ConnDx algorithm that patients should be asked whether (s)he experienced malaria episodes in the past 1–2 months and if so, microscopy should be prioritized.

The above-outlined challenges suggest diagnostics tests for febrile diseases, such as RDTs, should be embedded in a digital infrastructure of logistics and human decision support to raise to the next level of effectiveness and cost reductions. In the future, ConnDx could be deployed for bacterial infections if these can be diagnosed by RDTs (e.g. for C Reactive Protein), leading to better-informed antibiotic prescriptions, which is important in fighting antimicrobial resistance [45].

This study has several strengths and weaknesses. Strength is the important innovation of ConnDx providing reliable, geo-tagged and semi-real-time insights in malaria diagnostic and therapeutic services by private sector providers in a semi-rural setting in Kenya. Kisumu county hosts 94 private providers, which deliver approximately half of all primary healthcare services to its population (the government supplying care through 148 additional providers). With the majority (1 million) of Kisumu citizens currently connected to M-TIBA [46], ConnDx could in principle rapidly be scaled to all private providers and supplement the governments’ DHIS-2 database with valuable real-time private sector information. Another strength is the pioneering nature of this study that was supported by the local health authorities to run in parallel to existing malaria services. This allowed for rapid collection of important data, leading to actionable information for policymakers who demonstrated strong involvement.

In terms of weaknesses, this study was observational and not a formal clinical trial. Therefore, there are no statistically validated results on (improved) diagnostic performance and (improved) clinical outcomes for malaria. Moreover, in this study, there were no special provisions taken for febrile patients who tested negative for malaria and pertinent consequences concerning changes in clinical decision making. For example, it was not studied what the effect was of reduced malaria prescription on provider prescription of alternative drugs for fever (in particular, antibiotics) and what were the clinical consequences of such decisions. Furthermore, as the ConnDx process was not yet fully digitalized, several steps were still performed manually, such as linkage of cloud-databases and payout mechanisms. Therefore, the study did not allow for direct and automated feedback-loops with any of the participating stakeholders (patients, providers, payers, policymakers). For this reason, progress observed concerning quality improvement or increased cost-efficiency during this pilot was modest and likely due to the realization of providers that they were being remotely observed by the ConnDx intervention. There were also external factors that influenced the ConnDx pilot, such as civil unrest due to national elections, which hindered the uptake of participants due to security issues. Several strikes of medical staff put constraints on general malaria service provision. Moreover, M-TIBA is using Safaricom as the mobile operator (with a market share of 70% of Kenya), which became political in Kisumu where most of the population is from another tribal background than the Safaricom ownership, resulting in temporary boycotts of usage of this platform. Finally, it should be kept in mind that ConnDx was implemented in parallel to existing malaria services covered by the NHIF and the MoH. Thus, health providers could in principle benefit by participating in two parallel financing mechanisms, which could potentially create perverse incentives. This would not be the situation when ConnDx is fully integrated into NHIF or any other UHC prepayment mechanism and made a compulsory condition for payouts.

## Conclusions

This paper demonstrates the potential of ConnDx for more efficient malaria services at scale. ConnDx links important datasets in (semi) real-time, which previously were in silos and reported irregularly in DHIS-2. This allows for improved efficiencies at all levels of the healthcare system. For clients, the quality of care can improve by avoiding over-prescription of ineffective drugs and by providing the possibility to save and remunerate funds for malaria. Moreover, the linkage of patients’ telephone numbers to the platform allows for additional services like malaria information, appointment keeping, adherence support, patient feedback loops to providers on the experienced quality of care, individual alarms, early warning systems for geographic malaria hotspots, etc. For providers, better information is given on their diagnosis and treatment performance versus the National Guidelines, benchmarked and rated against colleagues. Moreover, providers save capitation fees when ConnDx is integrated into NHIF services, avoiding over-prescription of antimalarials. Finally, reputation will be increased due to better-quality care delivered. For payers, ConnDx reduces overhead costs by increasing transparency, supporting healthcare transactions at marginal costs. Funding can be traced to pertinent individual patient cases and vertical malaria funds can be integrated into larger UHC funding pools, covering both the public and private sector. For Kenyan policymakers and healthcare managers, ConnDx opens ample opportunities to timely identify weaknesses in service delivery and undertake targeted remedial actions, such as specific training to providers.

With ConnDx linking cloud-based databases of digital diagnostics data to a digital healthcare exchange platform such as M-TIBA, diagnostic results can target entitlements for malaria treatment directly through the mobile phones of M-TIBA users. This improved financial transparency, combined with marked quality gains through ConnDx presents a value proposition for scaling through (inter)national funders, such as the NHIF in Kenya, supported by the GFATM to channel vertical malaria funds through M-TIBA-facilitated payment platforms and contribute to UHC. ConnDx offers ample opportunities to enable more efficient service delivery for other high-morbidity medical conditions that can be digitally diagnosed, such as cervical cancer and cataract.

## Supplementary Information

**Additional file 1**. Interview guide qualitative interviews ConnDx.

## Data Availability

Due to privacy of patient data, datasets associated with this project are not publicly available. Anonymized data is available from the authors upon request.

## Abbreviations

ConnDx: Connected Diagnostics
DHIS-2: District Health Information Software
GFATM: Global Fund to Fight AIDS, Tuberculosis and Malaria
MoH: Ministry of Health
NHIF: National Hospital Insurance Fund
PMI: President's Malaria Initiative
SSA: Sub-Saharan Africa
UHC: Universal Health Coverage
WHO: World Health Organization

## References

[1] D'Acremont V (2009). Time to move from presumptive malaria treatment to laboratory-confirmed diagnosis and treatment in African children with fever. PLoS Med.

[2] Schroeder LF, Amukele T (2014). Medical laboratories in sub-Saharan Africa that meet international quality standards. Am J Clin Pathol.

[3] World Health Organization (WHO) (2014). A report on the misdiagnosis of HIV status.

[4] World Health Organization (WHO) (2010). Guidelines for the treatment of malaria—2nd edition.

[5] Yegorov S (2016). Low prevalence of laboratory-confirmed malaria in clinically diagnosed adult women from the Wakiso district of Uganda. Malar J.

[6] D'Acremont V (2014). Beyond malaria—causes of fever in outpatient Tanzanian children. N Engl J Med.

[7] World Health Organization (WHO) (2019). World malaria report 2019.

[8] Patouillard E (2017). Global investment targets for malaria control and elimination between 2016 and 2030. BMJ Glob Health.

[9] President's Malaria Initiative (PMI). 2019 [cited 2019 December, 31]. https://www.pmi.gov/.

[10] The Global Fund. 2019 [cited 2019 December, 31]. https://www.theglobalfund.org/en/.

[11] Wang J, et al. Preparedness is essential for malaria-endemic regions during the COVID-19 pandemic*.* The Lancet 2020. 10.1016/S0140-6736(20)30561-4 PMC7158917 32192582

[12] Plucinski MM (2015). Sleeping arrangements and mass distribution of bed nets in six districts in central and northern Mozambique. Trop Med Int Health.

[13] Afrane YA (2013). Utility of health facility-based malaria data for malaria surveillance. PLoS ONE.

[14] Oladosu OO, Oyibo WA. Overdiagnosis and overtreatment of malaria in Children that presented with fever in Lagos, Nigeria*.* ISRN Infectious Diseases 2013.

[15] Hume JC (2008). Household cost of malaria overdiagnosis in rural Mozambique. Malar J.

[16] Were V (2018). Socioeconomic health inequality in malaria indicators in rural western Kenya: evidence from a household malaria survey on burden and care-seeking behaviour. Malar J.

[17] Miorandi D, Sicari S, De Pellegrini F, Chlamtac I (2012). Internet of things: Vision, applications and research challenges. Ad Hoc Netw.

[18] Chutel L. Africa’s largest phone network is expanding its dominance with its own “smart feature phone. 2018 [cited 2019 December, 31]. https://qz.com/africa/1464244/africas-largest-phone-network-is-expanding-its-dominance-with-its-own-smart-feature-phone/

[19] World Databank. World development indicators—mobile cellular subscriptions 2017. 2017 [cited 2019 December, 31]. https://databank.worldbank.org/data/source/world-development-indicators

[20] Safaricom. Safaricom—MPESA. 2019 [cited 2019 December, 31]. https://www.safaricom.co.ke/personal/m-pesa

[21] M-TIBA Dashboard. 2019 [cited 2019 December, 31]. http://pharmaccess.m-tiba.org/home

[22] PharmAccess Foundation. HealthConnect. [cited 2019 December, 31]. https://www.healthconnectme.org/.

[23] Fio. Improve field performance and oversight of rapid diagnostic testing*.* [cited 2020 October 27]. http://fio.com/rapid-testing/

[24] i-calQ Technology [cited 2020 October 27]. https://i-calq.com/technology/

[25] Mobile assay homepage. [cited 2020 October 27]. https://mobileassay.com/

[26] Novarum™ DX. Homepage. [cited 2020, October 27]. https://bbisolutions.com/eu/services/reader-technology/novarum-dx.html

[27] Smith S (2019). Connected diagnostics: linking digital rapid diagnostic tests and mobile health wallets to diagnose and treat brucellosis in Samburu, Kenya. BMC Med Inform Decis Mak.

[28] Abdelhady R, Anan K, Elhussein A, Hussien MO, Mohammed EE, Elkhidir IM, Abdelhaleem A (2017). Prevelance of brucellosis among febrile negative malaria patients by PCR in Northern Kordofan State, Sudan. Clin Microbiol.

[29] KNBS (2019). 2019 Kenya population and housing census volume I: population by county and sub-county.

[30] Jenkins R (2015). Prevalence of malaria parasites in adults and its determinants in malaria endemic area of Kisumu County, Kenya. Malar J.

[31] Kisumu County*—Health at a glance*. 2015 [cited 2019 December, 31]. https://www.healthpolicyproject.com/pubs/291/Kisumu%20County-FINAL.pdf

[32] Garoup AC (2017). The malaria testing and treatment landscape in Kenya: results from a nationally representative survey among the public and private sector in 2016. Malaria J.

[33] WorldBank. Multiple Indicator Cluster Survey 2011, Nyanza Province. [cited 2019 December, 31]. https://microdata.worldbank.org/index.php/catalog/2660

[34] Atlas. *Qualitative data analysis*. [cited 2020 November, 7 ]; Available from: https://atlasti.com/.

[35] QGIS. A free and open source geographic information system. [cited 2020 November, 7 ]. https://qgis.org/en/site/index.html

[36] Kisumu monthly climate averages—rainfall and rain days. [cited 2020 February, 6]. https://www.worldweatheronline.com/kisumu-weather-averages/nyanza/ke.aspx

[37] Kenya malaria indicator survey 2015 2016, ICF international, Kenya National Bureau of Statistics Nairobi, Maryland

[38] Patel A (2012). Quality of generic medicines in South Africa: perceptions versus reality—a qualitative study. BMC Health Serv Res.

[39] Fadare JO (2016). The prescribing of generic medicines in Nigeria: knowledge, perceptions and attitudes of physicians. Expert Rev Pharmacoecon Outcomes Res.

[40] Kalogriopoulos NA (2009). Electronic medical record systems for developing countries: review. Conf Proc IEEE Eng Med Biol Soc.

[41] Dixit A (2016). Discovering the cost of care: consumer, provider, and retailer surveys shed light on the determinants of malaria health-seeking behaviours. Malar J.

[42] Ministry of Public Health and Sanitation, M.o.M.S. National guidelines for the diagnosis, treatment and prevention of malaria in Kenya*.* 2010.

[43] de Oliveira AM (2009). Performance of malaria rapid diagnostic tests as part of routine malaria case management in Kenya. Am J Trop Med Hyg.

[44] Proux S (2001). Paracheck-Pf: a new, inexpensive and reliable rapid test for *P. falciparum* malaria. Trop Med Int Health.

[45] Mekuria LA, de Wit TF, Spieker N, Koech R, Nyarango R, Ndwiga S, Fenenga CJ, Ogink A, Schultsz C, van’t Hoog A (2019). Analyzing data from the digital healthcare exchange platform for surveillance of antibiotic prescriptions in primary care in urban Kenya: A mixed-methods study. PLoS ONE.

[46] PharmAccess Foundation. Massive UHC Registration Drive to get underway in Kisumu County Kenya. 2018 [cited 2019 December, 31]. https://www.pharmaccess.org/update/massive-uhc-registration-drive-get-underway-kisumu-county-kenya/

