# Supplementary material for: Connected diagnostics to improve accurate diagnosis, treatment, and conditional payment of malaria services in Kenya
Source: BMC Med Inform Decis Mak. 2021 Aug 4;21:233. doi: 10.1186/s12911-021-01600-z (PMC8335459; doi:10.1186/s12911-021-01600-z)
Supplement: Supplementary file 1 — Additional file 1. Interview guide qualitative interviews ConnDx. [file 12911_2021_1600_MOESM1_ESM.pdf]

Interview guide

Clinic perspective on ConnDx

Confidential information PharmAccess, not to be shared.

Interview details

**Location of interview:**	…………………………………………	**Name of interviewee*:**	………………………………………………..
**Date of interview:**	…………………………………………	**Position title:**	………………………………………………..
**Interview no.:**	…………………………………………	**Age:**	………………………………………………..
		**Gender:**	M / F

**In the final report, statements of interviewees will be anonymous, and their real names will not be used*

Focus of the study

In 2018, Connected Diagnostics (ConnDx) was approved by local health authorities and piloted in seven health facilities in Kisumu County, testing over 12,500 patients total for malaria. For this reason, the objective of this study is to investigate different stakeholder perspectives on the implementation process of ConnDx in Kisumu, in particular from a providers' point of view. Findings derived from this project can be valuable for future improvements of the ConnDx services, as well as reporting back to policy makers and participating providers about outcomes to guide further improvement.

Interview preface

"First of all, I would like to thank you for allowing me to interview you. I am Shannen, and currently working on the Connected Diagnostic project a with a small team. This interview is based on the analysis we made of the data we collected last year during the Malaria Test&Treat campaign. During this interview, I would like to ask your opinion and reflection on the implementation of several aspects of Connected Diagnostics. This interview will be recorded, but your statements will remain completely anonymous. The interview will approximately take 30 minutes. Finally, if you have any questions during the interview, or if things are not clear, please let me know."

Clinic perspective on ConnDx - Consent to take part in research

(Read this out loud and ask the respondents if they 'agree')

This research was approved by the director of Kisumu County department of health. I would like to go over a few points in order to also get your consent for participating in this research by means of this interview.

First of all, participation in this study is voluntarily. It is possible for you to withdraw at any time or refuse to answer any question without any consequences. I explained the purpose and nature of this study to you, but if anything is unclear during this interview, there is an opportunity to ask questions. I would also like to mention that all information you provide for this study will be treated confidentially. In any report on the results of this research, your identity will remain anonymous. This will be done by changing for instance your name and disguising details of the interview. Extracts from your interview may be quoted in papers, reports, during discussions or in presentations. Furthermore, you are free to contact myself or any of the people involved in this study (from PharmAccess) to seek further clarifications and information if you want to. Finally, in order to analyse this interview later, this interview will be audio-recorded.

Do you understand and agree with all these points?

- ⇒ *If yes*, could you please say that you 'agree' for the recording?
- ⇒ *If not*, please do not hesitate to ask questions if something is not clear.

Provide contact information to respondents:

<u>Contact information</u>

Name interviewer: Shannen van Duijn
Email: shannenvanduijn@gmail.com

Names and emails of other people involved in this project:
Tobias Rinke de Wit, t.rinkedewit@pharmaccess.org
Emmanuel Milimo, e.milimo@pharmaccess.or.ke

1. Interview questions – Manager / owner of the clinic

Themes and questions	*Notes during interview*
I N T R O D U C T I O N	
How is diagnosis (and treatment) of patients done in the clinics in general?	
1) How long have you been working in this clinic?<br>2) Can you tell me something about your daily practices?<br>• What kind of tasks etc.<br><br>*Now in terms of malaria,*<br>3) Could you give an estimation of how many malaria patients come in the clinic on a weekly basis?	
What is currently done in [name of clinic] for malaria patients, other than testing and providing treatment?	
4) How do you feel about the role of this clinic in the prevention of malaria?<br>5) Are there currently any malaria outreach programs in place for communities that you are aware of?<br>6) Or any special programs for patients with co-morbidity?	
Procurement	
7) What are the practices on malaria drug supplies?<br>• Which types of anti-malarials are normally ordered (generic, branded)? Do you know the prices of these? Or can you give an estimation?<br>• Do you know who are your main suppliers of anti-malarials?<br>8) How do you think the business model shifted from (over)prescription of malaria drugs to malaria diagnostics?<br>• Does the private clinic receive reagents and drugs for free from the government or are procurements on the private market (sometimes) required?<br>• What do you think of the role of NHIF in this process?<br>• And the role of private insurers?	
(J U S T) B E F O R E C O N N D X	
9) When did you first starting using Connected Diagnostics?	

10) What was initially your reaction when you heard about this new way of working with (malaria) patients?
11) Do you remember what your expectations were about Connected Diagnostics before you started using it?
    - *Examples: beneficial for patients, time-saving, easy-to-use, complicated, new way of working etc.*

IMPLEMENTATION CONNDX

How did the clinics experience the ConnDx intervention? (How acceptable was it?)

Affective attitude

12) How do you feel about the usefulness of ConnDx in the health clinic?
    - What worked well and why?
13) What are your thoughts about M-TIBA (as carrier of ConnDx)?
    - Do you think its advantageous to use M-TIBA? Why?

Burden

14) During the period that you used ConnDx, were there any technical issues?
    - If yes, could you specify what kind of issues
    - Can you give an example of a case when that happened?
    - How did you handle the technical issues with the case?
15) Did you experience ConnDx as an extra effort on top of your daily tasks?
    - Why?
    - What exactly did you cost extra effort? (time, resources etc.)

Opportunity costs

16) In your opinion, what were the main benefits of ConnDx in the diagnosis and treatment of malaria patients?
17) In what way do you think the use of M-TIBA alongside ConnDx impacts administration and management?
    - *E.g. solves issues, gives an overview, faster and more clear administration etc.*

Perceived effectiveness

18) What, in your own words, is the main goal of the health clinic in treating malaria patients?
19) Do you think ConnDx contributed in achieving that goals?
    - Why/ why not?

• Example?	
F U T U R E C O N N D X	
What are the perspectives of the clinics regarding the future of ConnDx?	
Recommendations<br>*Since you have worked with ConnDx, and are familiar with it, I really value your input and feedback for the future of ConnDx.*<br><br>20) What recommendations would you give for health clinics or HCPs that will use ConnDx in the future?<br>21) Could you think of something that you would add to ConnDx in order to improve it?<br>22) What would you personally have done different during the implementation and process of ConnDx?	

2. Interview questions – Doctor/Nurse

Themes and questions	*Notes during interview*
INTRODUCTION	
How is diagnosis (and treatment) of patients done in the clinics in general?	
1) How long have you been working in this clinic?<br>2) Can you tell me something about your daily practices?<br>• What kind of tasks etc.<br>3) How do you typically deal with febrile illnesses?<br>• Can you maybe recall a case from last week from someone you treated who had a fever? How did you handle this? Could you walk me through the steps you took?<br>*Or*<br>• Imagine a child with fever comes into the health clinic and you help him. Could you walk me through the steps you would take from that point until the child is treated?<br><br>*Now in terms of malaria,*<br>4) Can you describe which steps in the process you consider important in the diagnosis of malaria patients?<br>• Why are these important to you?<br>5) Based on which criteria would you prescribe second line anti-malarials?<br>• Can you think of an example of a recent case in which you provided second line drugs? What symptoms did the patient have?<br>6) How often do you experience stock-outs of antimalarials?	
What is the role of the 'clinic reputation' in diagnosis?	
7) What is your view on using lab diagnostics always before prescribing drugs for a condition?<br>• How do you think it would impact quality of care, in comparison to presumptive treatment (i.e. treatment without lab diagnosis)?<br>• Do you think that a clinic could advertise with this? Why?<br>8) What is your opinion about the 'competition' by (semi-)legal drug sellers?	
(JUST) BEFORE CONNDX	

9) When did you first starting using Connected Diagnostics?

10) Do you remember what your expectations were about Connected Diagnostics before you started using it?
  - *Examples: beneficial for patients, time-saving, easy-to-use, complicated, new way of working etc.*

IMPLEMENTATION CONNDX

How did the clinics experience the ConnDx intervention? (How acceptable was it?)

Affective attitude

11) Can you give an example of how you used ConnDx to treat a malaria patient?
  - Can you think about the last time you used it?
  - What steps did you follow?

12) Do you feel your approach to dealing with febrile illness has changed since ConnDx implementation?
  - if so, how?

Intervention coherence

13) How long did you it take for you understand how ConnDx worked? (*e.g. several days, a week, more than a week*)

14) Was there anything unclear during the period you used ConnDx?
  - If yes, please specify which things
  - Did you receive enough support to get a better understanding?

Ethicality

15) How did you feel about using ConnDx with (malaria) patients?
  - How did patients generally react to ConnDx?
  - In your opinion, do you think ConnDx agreed or disagreed with your patients' values and how?
  - Did you experience any negative cases? Why? Example?

Burden

16) During the period that you used ConnDx, were there any technical issues?
  - For instance, with the reader?
  - If yes, could you specify what kind of issues

- Can you give an example of a case when that happened?
- How did you handle the technical issues with the case?

17) Did you experience ConnDx as an extra effort on top of your daily tasks?
    - Why?
    - What exactly did you cost extra effort? (time, resources etc.)

Opportunity costs

18) In your opinion, what were the main benefits of ConnDx in the diagnosis and treatment of malaria patients?
19) Can you also think of things (benefits, values) that you had to give up when you started using ConnDx?
    - Can you give an example?

Perceived effectiveness

20) What, in your own words, is the main goal of the health clinic in treating malaria patients?
21) Do you think ConnDx contributed in achieving that goals?
    - Why/ why not?
    - Example?

22) I would now like to go over a types of information ConnDx can collect. Could you please say for each point if you think it is of interest for the clinic in order to improve overall efficiencies? And why?
    - Patient flows (what type of patient comes from which geographic area and at what time)?
    - Incoming patient traffic to the clinic (?) and appointment systems?
    - Ratio 1st line drugs vs. 2nd line drugs?
    - Over-prescription percentage
    - Relation with seasons/patient flows?
    - Error-rates of lab RDT results?
    - Socio-economic indicators of patients/customers?
    - Use of branded versus generic drugs?
23) What additional types of information do you feel would be valuable for ConnDx to collect

FUTURE CONNDX	
What are the perspectives of the clinics regarding the future of ConnDx?	
Self-efficacy<br>24) Do you feel confident to continue using ConnDx in the future?<br>• Why/why not?<br><br>*Recommendations*<br>*Since you have worked with ConnDx, and are familiar with it, I really value your input and feedback for the future of ConnDx.*<br><br>25) What recommendations would you give for health clinics or HCPs that will use ConnDx in the future?<br>26) Could you think of something that you would add to ConnDx in order to improve it?	

3. Interview questions – Administrative person (receptionist)

Themes and questions	*Notes during interview*
INTRODUCTION	
How is diagnosis (and treatment) of patients done in the clinics in general?	
1) How long have you been working in this clinic?<br>2) Can you tell me something about your daily practices?<br>• What kind of tasks etc.<br><br>*Now in terms of malaria,*<br>3) Could you give an estimation of how many malaria patients come in the clinic on a weekly basis?<br>4) How are malaria data currently administered in the facility?<br>• *Paper files, digital files, lab records, dispensary records, clinical records, admin records?*	
What is currently done in [name of clinic] for malaria patients, other than testing and providing treatment?	
5) How do you feel about the role of this clinic in the prevention of malaria?<br>6) Are there currently any malaria outreach programs in place for communities that you are aware of?<br>7) Or any special programs for patients with co-morbidity?	
(JUST) BEFORE CONNDX	
8) When did you first starting using Connected Diagnostics?<br>9) What was initially your reaction when you heard about this new way of working with (malaria) patients?<br>10) Do you remember what your expectations were about Connected Diagnostics before you started using it?<br>• *Examples: beneficial for patients, time-saving, easy-to-use, complicated, new way of working etc.*	
IMPLEMENTATION CONNDX	
How did the clinics experience the ConnDx intervention? (How acceptable was it?)	
Affective attitude<br>11) How do you feel about the usefulness of ConnDx in the health clinic?<br>• What worked well and why?	

12) What are your thoughts about M-TIBA (as carrier of ConnDx)?
    - Do you think its advantageous to use M-TIBA? Why?

Intervention coherence

13) How long did you it take for you understand how ConnDx worked? (*e.g. several days, a week, more than a week*)
14) Was there anything unclear during the period you used ConnDx?
    - If yes, please specify which things
    - Did you receive enough support to get a better understanding?

Burden

15) During the period that you used ConnDx, were there any technical issues?
    - If yes, could you specify what kind of issues
    - Can you give an example of a case when that happened?
    - How did you handle the technical issues with the case?
16) Did you experience ConnDx as an extra effort on top of your daily tasks?
    - Why?
    - What exactly did you cost extra effort? (time, resources etc.)

Opportunity costs

17) In your opinion, what were the main benefits of ConnDx in the diagnosis and treatment of malaria patients?
18) In what way do you think the use of M-TIBA alongside ConnDx impacts administration and management?
    - *E.g. solves issues, gives an overview, faster and more clear administration etc.*

Perceived effectiveness

19) I would now like to go over a types of information ConnDx can collect. Could you please say for each point if you think it is of interest for the clinic in order to improve overall efficiencies? And why?

- Patient flows (what type of patient comes from which geographic area and at what time)?
- Incoming patient traffic to the clinic (?) and appointment systems?
- Ratio 1st line drugs vs. 2nd line drugs?
- Over-prescription percentage
- Relation with seasons/patient flows?
- Error-rates of lab RDT results?
- Socio-economic indicators of patients/customers?
- Use of branded versus generic drugs?

20) What additional types of information do you feel would be valuable for ConnDx to collect

FUTURE CONNDX

What are the perspectives of the clinics regarding the future of ConnDx?

Recommendations

Since you have worked with ConnDx, and are familiar with it, I really value your input and feedback for the future of ConnDx.

21) Could you think of something that you would add to ConnDx in order to improve it?

22) What would you personally have done different during the implementation and process of ConnDx?

4. Interview questions – Lab tech

Themes and questions	*Notes during interview*
INTRODUCTION	
How is diagnosis (and treatment) of patients done in the clinics in general?	
1) How long have you been working in this clinic?<br>2) Can you tell me something about your daily practices?<br>• What kind of tasks etc.<br><br>*Now in terms of malaria,*<br>3) Could you give an estimation of how many malaria patients come in the clinic on a weekly basis?<br>4) Can you describe which steps in the process you consider important in the diagnosis of malaria patients?<br>• Why are these important to you?<br>5) Under which circumstances would you use RDT to test for malaria instead of microscopy?<br>6) What is the procedure on quality checking of anti-malarials (active ingredients)?	
What is the role of the 'clinic reputation' in diagnosis?	
7) What is your view on using lab diagnostics always before prescribing drugs for a condition?<br>• How do you think it would impact quality of care, in comparison to presumptive treatment (i.e. treatment without lab diagnosis)?<br>• How do you think it would influence the competitiveness of your clinic? E.g.<br>○ Competition for patients<br>○ Competition for funding<br>• Do you think that a clinic could advertise with this? Why?<br>8) What is your opinion about the 'competition' by (semi-)legal drug sellers?	
IMPLEMENTATION CONNDX	
How did the clinics experience the ConnDx intervention? (How acceptable was it?)	
Affective attitude<br>9) How do you feel about the usefulness of ConnDx in the health clinic?<br>• What worked well and why?	

10) Do you prefer using RDT for malaria testing over microscopy?<br>• Why or why not?<br>• How would you feel if the clinic becomes less dependent on microscopy, electricity and skilled lab tech for malaria testing?	
Intervention coherence<br>11) How long did you it take for you understand how ConnDx worked? (*e.g. several days, a week, more than a week*)<br>12) Was there anything unclear during the period you used ConnDx?<br>• If yes, please specify which things<br>• Did you receive enough support to get a better understanding?	
Burden<br>13) During the period that you used ConnDx, were there any technical issues?<br>• For instance, with the reader?<br>• If yes, could you specify what kind of issues<br>• Can you give an example of a case when that happened?<br>• How did you handle the technical issues with the case?<br>14) Did you experience ConnDx as an extra effort on top of your daily tasks?<br>• Why?<br>• What exactly did you cost extra effort? (time, resources etc.)	
Opportunity costs<br>15) In your opinion, what were the main benefits of ConnDx in the diagnosis and treatment of malaria patients?<br>16) Can you also think of things (benefits, values) that you had to give up when you started using ConnDx?<br>• Can you give an example?	
Perceived effectiveness<br>17) I would now like to go over a types of information ConnDx can collect. Could you please say for each point if you think it is of interest for the clinic in order to improve overall efficiencies? And why?<br>• Patient flows (what type of patient comes from which geographic area and at what time)?	

- Incoming patient traffic to the clinic (?) and appointment systems?
- Ratio 1st line drugs vs. 2nd line drugs?
- Over-prescription percentage
- Relation with seasons/patient flows?
- Error-rates of lab RDT results?
- Socio-economic indicators of patients/customers?
- Use of branded versus generic drugs?

18) What additional types of information do you feel would be valuable for ConnDx to collect?

FUTURE CONNDX

What are the perspectives of the clinics regarding the future of ConnDx?

Self-efficacy

19) Do you feel confident to continue using ConnDx in the future?
    - Why/why not?

Recommendations

Since you have worked with ConnDx, and are familiar with it, I really value your input and feedback for the future of ConnDx.

20) What recommendations would you give for health clinics or HCPs that will use ConnDx in the future?
21) Could you think of something that you would add to ConnDx in order to improve it?
22) What would you personally have done different during the implementation and process of ConnDx?

Closing of the interview

"This is the end of the interview. Thank you so much for your answers. Is there anything else that you want to tell me that might be relevant for the research project? If you think of something relevant later that you are willing to share, I provided my personal details to you, so feel free to contact/email me. Additionally, do you have any other questions that you want to ask?

Then, I want to thank you for your time and wish you a very nice day/weekend"

The end of the interview guide
